# Bilateral transcranial direct current stimulation may be a feasible treatment of Parkinsonian tremor

**DOI:** 10.3389/fnins.2023.1101751

**Published:** 2023-02-24

**Authors:** Bin Zhang, Feifei Huang, Jun Liu, Dingguo Zhang

**Affiliations:** ^1^State Key Laboratory of Mechanical Systems and Vibrations, Robotics Institute, Shanghai Jiao Tong University, Shanghai, China; ^2^Department of Neurology, Rui Jin Hospital, School of Medicine, Shanghai Jiao Tong University, Shanghai, China; ^3^Department of Electronic and Electrical Engineering, University of Bath, Bath, United Kingdom

**Keywords:** transcranial direct current stimulation, Parkinson's disease, Parkinsonian tremor, tremor suppression, electrode setups

## Abstract

**Background:**

Parkinsonian tremor is a common pathological tremor that affects over 6 million people worldwide. It lowers patients' quality of life and threatens their career development, especially when patients' occupation requires dexterous manipulation. In spite of current available treatments in clinics, there is a lack of low-cost, low side-effect, effective solutions for Parkinsonian tremor. Transcranial direct current stimulation (tDCS) may be an alternative treatment.

**Objective:**

In this research, we explored the immediate effect of tDCS with a novel bilateral electrode setup over Parkinsonian tremor. In such a bilateral setup, the cathode was placed over the primary cortex contralateral to the more affected side of Parkinsonian tremor while the anode symmetrically over the other hemisphere. It was designed as a modification to the traditional cathodal setup. The performance of this bilateral setup was compared with three other setups including anodal setup, cathodal setup, and sham (control).

**Methods:**

A randomized, sham-controlled, double-blind, crossover experiment was carried out over 13 qualified patients diagnosed with idiopathic Parkinson's disease (PD). Before and after the stimulus of each tDCS setup, subjects were tested before and after tDCS with four measures, including the Unified Parkinson's Disease Rating Scale (UPDRS), Fahn-Tolosa-Marin Tremor Rating Scale (FTMTRS), Purdue Pegboard Test (PPT) and a self-design Continuous Tremor Signal Assessment (CTSA). Tremor intensity calculated from CTSA data were regarded as the primary outcome of the experiment.

**Results:**

Statistical results of CTSA, FTMTRS and PPT showed both bilateral tDCS and cathodal tDCS effectively suppressed Parkinsonian tremor. A quantitative comparison of the effect in tremor suppression indicated the optimal suppressive effect was obtained with bilateral tDCS. Based on the results of UPDRS, anodal tDCS was found to benefit subjects' overall performance the most, however, it had little effect in improving Parkinsonian tremor, as revealed by the results of other evaluations.

**Conclusion:**

Our study suggests a beneficial immediate effect of bilateral tDCS in Parkinsonian tremor suppression. In addition, we assume there may be an underlying interhemispheric unbalance of cortical excitability which contributes to Parkinsonian tremor genesis.

**Clinical trial registration:**

Identifier: ChiCTR2100054804.

## 1. Introduction

Pathological tremor is the most common movement disorder that manifests as an involuntary, large-amplitude, rhythmic oscillation in human body (Helmich et al., 2013). It lowers patients' quality of life by affecting their daily behaviors (Berk et al., 2002). Particularly by weakening limb control ability (especially fine movements), tremor can threaten patients' career development especially when patients' occupation requires dexterous manipulation (Dick et al., 2000; Lee et al., 2014). Parkinsonian tremor is an early unfatal symptom originated from Parkinson's disease (PD) (Deuschl et al., 1998). However, as time moves, it will deteriorate in intensity alongside with other complications (Weintraub and Stern, 2005; Tinazzi et al., 2006) and finally lead to subsequent fatal accidents, such as falls (Gray and Hildebrand, 2000). As indicated by a recent investigation related with PD patients, Parkinsonian tremor tremendously reduced patients' life satisfactory level (Rosqvist et al., 2017). To tackle this, a timely effective treatment is needed once Parkinsonian tremor takes place.

The most fundamental therapy targeting Parkinsonian tremor is pharmaceutical treatment. Although easy to access (levodopa, dopamine agonists etc.), it has been constantly reported with unsatisfactory side-effects as well as the weakening treatment effect post honeymoon (Rinne, 1983; Borovac, 2016; Nonnekes et al., 2016). Moreover, there is significant drug action difference between individuals, which may lead to suboptimal clinical prescription (Pavese et al., 2006; Tomlinson et al., 2010). Regardless of pharmaceutical treatments, deep brain stimulation (DBS) (Benabid, 2003), representative of relevant surgical operations, is mainly considered for the levodopa-responsive patients with intermediate symptoms of PD (Bronstein et al., 2011). It facilitates a reduction of dopamine absorption amount (Kleiner-Fisman et al., 2006) and substantially improve the quality of life with Parkinsonian tremor by decreasing the tremor intensity (Diamond and Jankovic, 2005). However, patients have to bear high cost for the surgical operation along with high risks of major surgery (hemorrhage, infection, hallucination, severe depression etc.) (Doshi, 2011). With regard to other available surgical treatments, pallidotomy and thalamotomy are little considered because of their irreversible lesion to the brain (Lee et al., 2018). Other novel attempts to offset Parkinsonian tremor with antagonist muscles includes methods applying functional electrical stimulation (FES) (Maneski et al., 2011; Zhang et al., 2011). However, it was withheld from practical use due to electric safety concern where electricity was applied directly to human skin surface.

Considering neuromodulation, there have been some promising, non-invasive neuromodulation techniques. One of them is transcranial direct current stimulation (tDCS) which elicits sustained cortical excitability alterations by inducing low-intensity direct currents onto the scalp (Nitsche et al., 2008). Compared to other neuromodulation techniques such as transcranial magnetic stimulation (TMS) that requires bulky and expensive device, tDCS requires much lower cost device and is more portable to be home-used (Hallett, 2007). Therefore, considering practical conditions, tDCS may better suit the vast and urgent need of patients suffering from Parkinsonian tremor. A quantity of studies investigating tDCS have been carried out and confirmed the efficacy of tDCS in various applications, including cognitive enhancement (Coffman et al., 2014), and disease treatments, such as depression treatment (Fregni et al., 2006a). Targeting incurable neurologic disorders, tDCS was first used in stroke. A study by Fregni et al. (2005) found out tDCS could significantly improve the motor functions of stroke patients with either anodal stimulus over their affected primary cortex or cathodal stimulus over their unaffected primary cortex. The effect of tDCS was also proven by other studies combining tDCS with other methods, such as tDCS + occupational therapy (Nair et al., 2011), tDCS + FES (Shaheiwola et al., 2018) etc. Additionally, the motor functions of stroke patients were found to improve better with combined methods than with either technique alone. Fundamental studies demonstrated the effect of tDCS is polarity-dependent: in general, anodal tDCS (a-tDCS) facilitates cortical excitability while cathodal tDCS (c-tDCS) inhibits (Nitsche and Paulus, 2000, 2001). A study by Mahmoudi et al. (2011) proposed a novel setup of electrode placement named bilateral tDCS, where two pairs of electrodes were used together. Their results showed bilateral tDCS induced more motor improvement in stroke patients than traditional setups. The effect of bilateral tDCS in motor cortex excitability alteration was proven by another study by Di Lazzaro et al. (2014) with motor evoked potentials (MEP).

The application of tDCS in Parkinson's disease was initiated by Fregni et al. (2006b). According to their MEP results, both anodal tDCS and cathodal tDCS elicited an immediate motor cortex excitability alteration after stimulus; however, UPDRS results showed only anodal tDCS facilitated a significant improvement in PD patients' comprehensive motor function. The lasting effect of tDCS in PD was demonstrated by Benninger et al. (2010) who conducted an 8-session anodal tDCS over a cohort of qualified PD patients. Their results showed long-term tDCS treatment could especially improve PD symptoms of bradykinesia and mobility. In addition, tDCS treatment was found to affect PD patients in working memory (Boggio et al., 2006), functional mobility (Manenti et al., 2014), freezing of gait (Valentino et al., 2014), executive function (Doruk et al., 2014) etc. Attempts to enhance the effect of tDCS were made by combining tDCS with other methods, such as gait training (Costa-Ribeiro et al., 2017; Fernández-Lago et al., 2017), dancing (Kaski et al., 2014) and cognitive training (Manenti et al., 2018). While most studies evaluated the effect of tDCS with scales targeting the comprehensive motor function behaviors of PD, few has focused on Parkinsonian tremor.

In our research, we investigated the immediate effect of tDCS with different electrode placement setups and focused on Parkinsonian tremor which occurs in upper limbs. A novel bilateral electrode setup was considered (bilateral tDCS, b-tDCS) where a pair of anode and cathode was placed over the scalp symmetrically. It was designed as a modification of anodal setup. We expected b-tDCS could produce a similar or better effect in suppressing Parkinsonian tremor. This bilateral setup was never used for PD, however has been tested to be safe in studies of stroke treatment. Related to PD, we found only one study investigating balance and fear of fall, where bilateral anodal tDCS was applied and two pairs of electrodes were used (Hadoush et al., 2018). Thus, to our knowledge, the bilateral setup used in our research was the first time to be applied in the topic of PD. As comparison, traditional anodal and cathodal setup (a-tDCS and c-tDCS), along with sham tDCS (s-tDCS) as control, were considered in the experiment. In addition, primary motor cortex (M1) was chosen as the target area for stimulation in accordance with previous studies (Fregni et al., 2006b; Benninger et al., 2010).

## 2. Methods

### 2.1. Subjects

**Figure 1:**
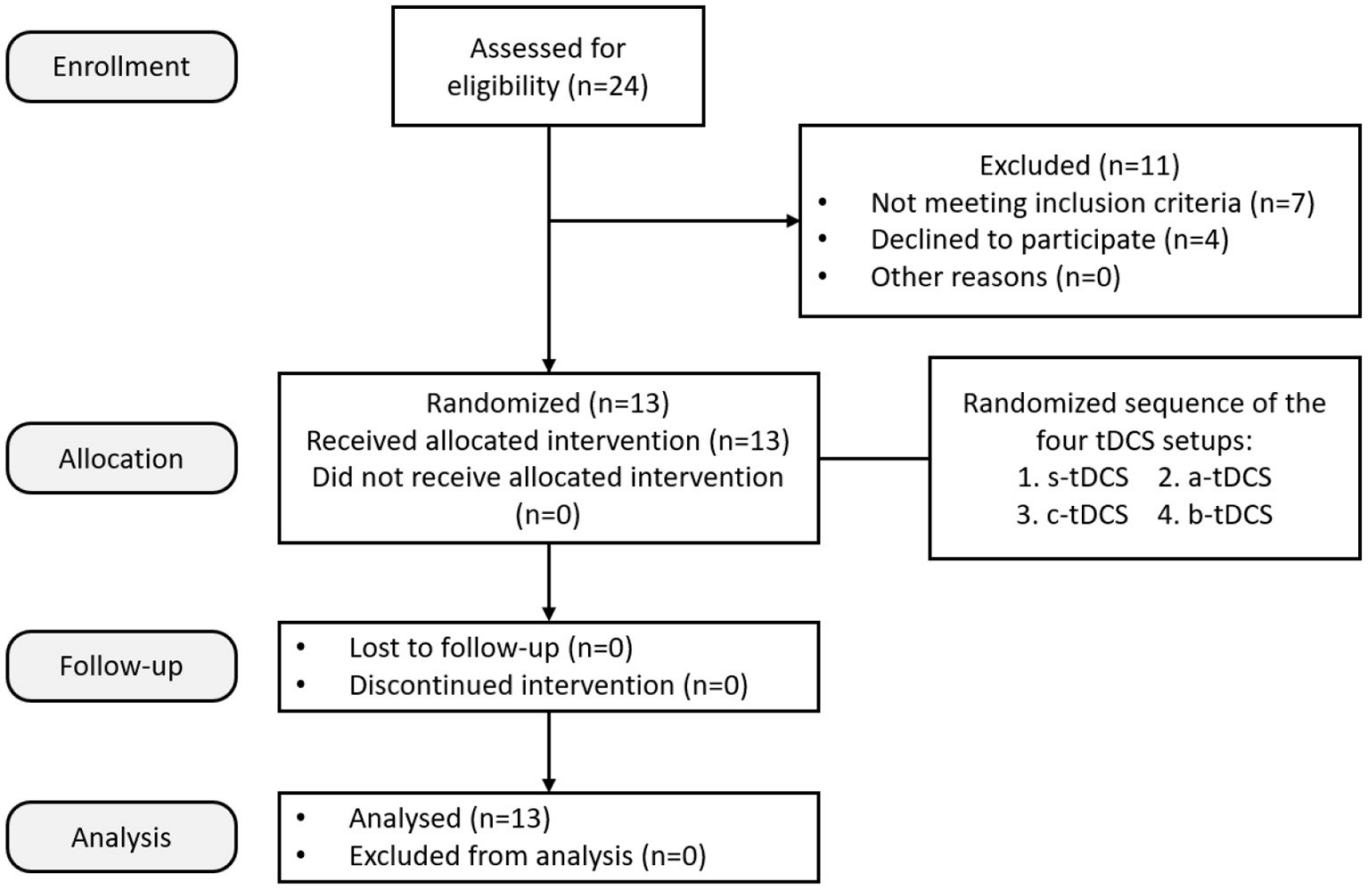
CONSORT diagram demonstrating the study recruitment process.

The research has been approved by the Chinese Clinical Trial Registry (Registration No.: ChiCTR2100054804) and the local Ethics Committee of Shanghai Jiao Tong University, China (Approved No. of Ethic Committee: 2019 Clinical Trial No. 136). All subjects provided written consent after being informed of the purpose and the procedures of the experiment. The overall experiment was strictly performed in accordance with all relevant guidelines and regulations of the institutional review board and the Declaration of Helsinki. Patients were recruited by the department of neurology of Rui Jin Hospital (Shanghai, China). All of them were out-patients with upper limb tremor resulted from Parkinson's disease (Excluded *N* = 11; Not meeting the inclusion criteria *N* = 7; Declined to participate *N* = 4) (Figure 1).

The inclusion criteria for subjects were: (1) age between 50 and 80 years old, (2) confirmed diagnosis of idiopathic Parkinson's disease according to the MDS clinical diagnostic criteria for PD (Postuma et al., 2015), (3) symptom of upper limb tremor (rest tremor or postural tremor) resulted from PD, and (4) modified Hoen & Yahr (H&Y) Stage 1 to 3 (Hoehn and Yahr, 1967). The exclusion criteria included: (1) history of other diseases that may lead to pathological tremor, such as essential tremor (Deuschl et al., 1998), (2) under the treatment of other neuromodulation therapy, such as DBS, within recent 1 month, (3) history of mental problems, including anxiety, dementia, hallucination or delusion etc., (4) strong reliance on anti-Parkinson medications, or (5) history of cognitive disorder [Mini-mental State Examination (MMSE) score ≤ 16] (Tombaugh and McIntyre, 1992).

The average disease duration of PD among all subjects [7M/6F, all right-handed, aged 67.5 ± 4.9 (mean ± SD)] were 4.38 ± 1.86 (mean ± SD) years. The Unified Parkinson's Disease Rating Scale (UPDRS) score ranged from 20 to 78 [31.7 ± 15.4 (mean ± SD)] while the modified H&Y stage ranged from 1.0 to 3.0 [1.8 ± 0.8 (mean ± SD)]. The tremor was found to be lateralized in all subjects. Throughout the manuscript, we refer to the more-affected side (MAS) as the side of body exhibiting more severe tremor, which was determined visually by an experienced physician, while the less-affected side (LAS)was defined as the other. Of all subjects, about half (*n* = 7) were found to be more-affected by tremor on the left side with the other half (*n* = 6) on the right side. The average levodopa-equivalent daily dose (LEDD) among all subjects was 278.8 ± 203.3 (mean ± SD) mg according to the calculation protocol provided by Tomlinson et al. (2010). In order to exclude drug effects, all subjects were told to discontinue anti-Parkinson medications on the day of the experiment, which ensured a withdrawal period of more than 12 h. Anti-Parkinson medications were resumed immediately after the experiment session of the day.

### 2.2. Study design

**Figure 2:**
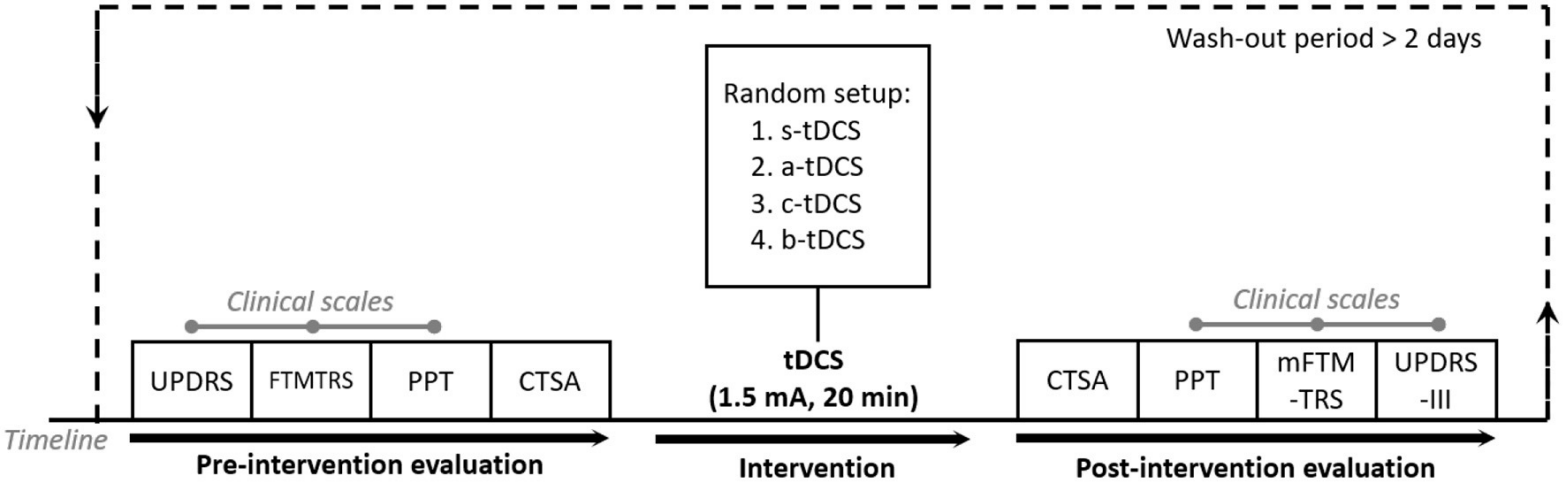
Experimental protocol.

The experiment followed a randomized, sham-controlled, double-blind, crossover design (Schulz et al., 2010). It was designed based on the baseline stability assumption that the subjects could maintain a constant tremor manifestation if left unaffected. The aim of this experiment is to investigate the short-term (immediate) effect over Parkinsonian tremor, with three different active tDCS setups, namely the anodal, cathodal and bilateral setup. In order to exclude placebo effects, a control setup with sham tDCS was considered for comparison. Therefore, the complete experiment consisted of four different setups, with each corresponding to a session in the experiment (Figure 2). The sequence of carrying out the four sessions over each subject was generated by a random sequence generator programmed on Matlab R2015b (Mathworks Inc., USA). Each session was conducted around the same time of the day to minimize the circadian influences. It started with the pre-intervention evaluations involving three clinical scales and the continuous tremor signal assessment (CTSA), followed immediately by the tDCS intervention (sham/active) and the post-intervention evaluation. During the experiment, a specific physician served as the evaluator and finished all evaluations, while another experimenter performed the tDCS intervention. All subjects and the physician were blind to the current tDCS setup. Between each session, there was a long enough wash-out period of more than 2 days to clear up the effect of the previous intervention. In subsequent analysis, we evaluated the effect of the current setup of tDCS by comparing the performance of subjects before and after the tDCS intervention.

The three related clinical scales were: (1) UPDRS, (2) Fahn-Tolosa-Marin Tremor Rating Scale (FTMTRS) and (3) Purdue Pegboard Test (PPT). To lessen the time cost of the evaluation, full UPDRS and full FTMTRS were only used as a pre-intervention evaluation and a simplified version of them, namely the simplified version with only Part III of the UPDRS that relates motor function (UPDRS-III) and the modified FTMTRS (mFTMTRS) comprising Part A and Part B in FTMTRS, was used as the post-intervention evaluation. The assessment of the PPT remained the same before and after the intervention. To obtain more accurate and detailed description of tremor, we designed the procedure of CTSA where tremor acceleration and EMG signals were assessed. The sensors used for the CTSA were kept on subjects until the end of the post-intervention CTSA. In case that the accelerometer and EMG sensors might interfere with subjects' performance in chosen scales, we modified the sequence of the measures and arranged CTSA to be the last pre-intervention evaluation and the first post-intervention evaluation.

### 2.3. Intervention

**Figure 3:**
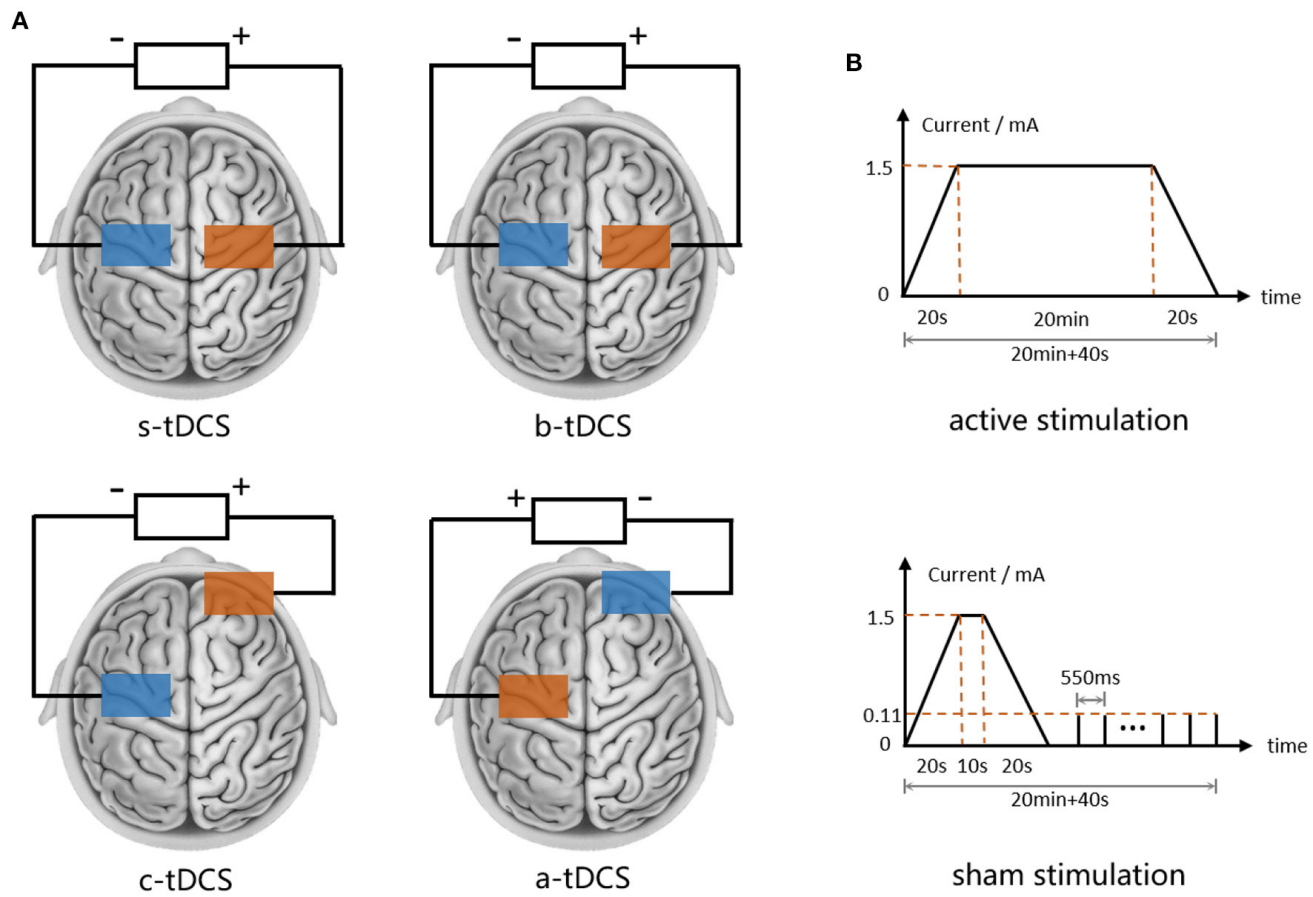
tDCS settings. **(A)** Electrode placement of s-tDCS, b-tDCS, c-tDCS and a-tDCS. **(B)** Current waveform of active and sham tDCS.

To apply tDCS, a commercial CE-certified device named DC-Stimulator (NeuroConn GmbH, Germany) was used. For each subject, we started by locating the primary motor cortex (M1) on the more-affected side through targeting the abductor pollicis brevis (APB) hot spot at rest with transcranial magnetic stimulation (TMS) with a device called Magstim Rapid 2 (Magstim Co., UK). A pair of sponge electrodes (6.5 cm*6.5 cm) moistened with 0.9% NaCl solution were placed regarding different tDCS setups, as shown in Figure 3A (assuming the MAS is on subject's left side): in the anodal setup, the anode was placed over the left M1 hotspot and the cathode was placed over the right supraorbital region. An opposite electrode placement setup was used in the cathodal setup. For bilateral setup, we placed the anode and the cathode over the right and the left M1 hotspot symmetrically. In all aforementioned active stimulations, a direct current of 1.5 mA was delivered constantly to the skull over 20 min with a ramp-up and ramp-down of 20 s. The parameters of the active stimulations were chosen in accordance with the most up-to-date safety guidelines for tDCS (Bikson et al., 2016). In sham tDCS, the electrode placement was the same as in the bilateral setup, however, in the 20-min protocol the direct current only lasted for a short time, followed by a serial pulse train of 110 uA (Figure 3B) without any therapeutic effect (Palm et al., 2013). In either active or sham setup of tDCS, the subject was seated comfortably on a chair in a quiet state and waited until the end of the intervention.

### 2.4. Continuous tremor signal assessment

In order to quantify upper limb tremor for comparison, we collected two continuous tremor signals, which were tremor acceleration and EMG signals, respectively. Before experiment, the tremor type (postural tremor/resting tremor) to record of each subject was decided independently by a physician after patient's enrollment. The main principle of choosing the main tremor type is tremor intensity and stability. Intentional tremor was not considered in our research since it is difficult to standardize motion. For subjects whose main tremor type is resting tremor, we had them seated comfortably with arms fully supported on armrests and recorded their tremor. For the others, postural tremor was inspected with seated subject stretching the whole upper limb forward and maintaining the posture for some time (Zhang et al., 2018). Additional requirements in recording postural tremor included: (1) fingers closed, (2) palms facing downward, and (3) seated upright.

All data was recorded through a commercial device system named the Biometrics Datalog (Biometrics Inc., the USA), along with a three-axis accelerometer sensor and four surface EMG (sEMG) sensors. The accelerometer sensor was fixed onto the third knuckle of the middle finger on the more affected side. Four sEMG sensors were attached respectively onto the muscle bellies of the flexor carpi radialis (FCR), the flexor carpi ulnaris (FCU), the extensor carpi radialis (ECR) and the extensor carpi ulnaris (ECU). The data of tremor acceleration and EMG signals were both digitized into 1,000 Hz and simultaneously recorded. From each subject, we obtained a 5-min sequential data package comprising tremor acceleration and EMG signals.

### 2.5. Clinical measures

The full UPDRS is a standardized evaluation test for both motor and non-motor deficits of Parkinson's disease (Goetz et al., 2008) and was utilized in this experiment to assess the baseline of subjects. Since this research mainly focused on the symptom of tremor, a subscale consisting of Part III in UPDRS (UPDRS-III, Motor Examination) was used instead after intervention. The excluded parts were: (Part I) Non-motor Experiences of Daily Living, (Part II) Motor Experiences of Daily Living and (Part IV) Motor Complications, which were less associated with the immediate response of tremor.

FTMTRS is a tool used to evaluate tremor severity in human body (Fahn et al., 1993). The full scale was used in the pre-intervention evaluation to assess baseline. A subscale comprising Part A and Part B in FTMTRS was used as a corresponding post-intervention evaluation. We excluded Part C: Functional Disabilities Resulting from Tremor for it had less relevance with the short-term response in tremor.

PPT is a tool for manipulative dexterity evaluation and requires the subject to place the specific small objects as many as possible in the limited time (Tiffin and Asher, 1948). Since upper limb tremor was one of the factors that affect subject's hand dexterity, we assumed any change in subject's performance in PPT manifested the change in tremor severity. The scale consists of four parts: (1) moving pins with the right hand, (2) moving pins with the left hand, (3) moving pins with both hands and (4) assembling pins, collars and washers with both hands. We followed the standard procedure to implement PPT over subjects. Subjects were instructed to practice the tasks before the evaluation and afterwards underwent a three-trial PPT in both the pre- and post-evaluation.

### 2.6. Data processing

The raw scores of the clinical scales were summarized in Excel (2015, Microsoft Corp., the USA). For UPDRS-III, we computed: (1) the sum score of the UPDRS-III and (2) the sum score of item 20 and item 21 in UPDRS-III (UPDRS-III-tr). Please note that item 20 and item 21 in UPDRS III are the items that evaluates the intensity of upper limb resting tremor and postural tremor. For mFTMTRS, we computed: (1) the sum score of the mFTMTRS, (2) the sum score of the items related to the more-affected-side tremor (mFTMTRS-MAS) and (3) the sum score of the items related to the less-affected-side tremor (mFTMTRS-LAS). Assuming the more affected side was the right side of the body, the items related to mFTMTRS-MAS were item 5, 8, 11-Right, 12-Right, 13-Right, 14-Right, and 15-Right while items related to mFTMTRS-LAS were item 6, 8, 11-Left, 12-Left, 13-Left, 14-Left, and 15-Left. For PPT, the score of each item was first averaged among the 3 trials of PPT. We then computed: (1) the sum score of the PPT, (2) the score related to the more affected hand (PPT-MAS) and (3) the score related to the less affected hand (PPT-LAS). Assuming the more affected side was the right side, the score related to the more affected hand was the score of part 1, and the score related to the less affected hand was that of part 2.

The sequential data of the CTSA was converted into its text format and processed in Matlab (R2017a, MathWorks Inc., the USA). For three-axis tremor acceleration signals, the z-axis data that incorporated the most information of tremor (perpendicular to the ground) was used for analysis. A second-order zero-phase Butterworth bandpass filter with a passband from 0.5 Hz to 10 Hz was applied over the z-axis data to remove the zero-shifting of hardware sampling, the low-frequency voluntary movement components and the high-frequency irrelevant components (e.g., noise). For ease of further calculation, the filtered acceleration signal was then down-sampled to 100 Hz. For EMG signals, we applied a second-order zero-phase Butterworth bandpass filter with a passband from 0.5 Hz to 450 Hz to preserve the significant components of EMG activities (Merletti and Di Torino, 1999). A denoising notch filter was then utilized to remove the 50-Hz power line interference and its higher harmonics. Among the four EMG channels, the one with the largest mean absolute value (MAV) was selected for further analysis.

Both filtered acceleration and EMG signals were subsequently characterized in amplitude, frequency and shape. To feature amplitude, we computed root mean square (RMS) value as the primary outcome (Equation 1).


(1) $$\text{R}MS=\sqrt{\frac{1}{N}\sum_{i=1}^{N}x_{i}^{2}}$$

where *x*_*i*_ is the *i*-th sample in the data sequence (*i* = 1, 2, …, *N*).

In addition, the filtered acceleration data of each subject was segmented with a 1-second non-overlap sliding window, after which a set of each subject consisting of 300 acceleration segments could be generated. For each subject, we computed the RMS value of each acceleration segment and generated the set of segmented RMS (sRMS) value (Equation 2).


(2) $$\text{sRMS}=\{\text{RMS}(X_{1}),\text{RMS}(X_{2}),\ldots ,\text{RMS}(X_{N_{X}})\}$$

where *X*_*j*_ ∈ *A* is the *j*-th segment of set *A* (*j* = 1, 2, 3, …, *N*_*X*_).

The sRMS sequence was then sorted from small to large and generated another sequence called sorted sRMS (ssRMS) sequence. The ssRMS sequence manifested the distribution of sub-regional tremor amplitude. For ease of description, the ssRMS values were labeled from 1 to 300 based on their numerical order in the ssRMS sequence. The maximum value in the ssRMS sequence was also marked down as a feature for tremor acceleration.

To characterize the filtered tremor acceleration sequence in frequency, we computed the dominant frequency of its bispectrum. Bispectrum transform was chosen because it is mathematically able to describe non-linear, non-Gaussian, stochastic signals, such as Parkinson's tremor signals. It yields features that are more stable and anti-noise compared to those generated from power spectrum density (PSD) (Zhang et al., 2018). Here in this research, we computed dominant frequency by tracking the peak frequency on the diagonal slice of bispectrum. For EMG signals, we computed the feature called zero-crossing (ZC) to represent frequency, as shown in Equation 3.


(3) $$\begin{array}{l} \text{ZC}=\overset{N}{\underset{i=1}{\sum }}\text{s}gn\left(-x_{i}x_{i+1}\right) \end{array}$$

where $\text{s}gn(x)=\{\begin{array}{cc} 1 & x>0\\ 0 & \text{otherwise} \end{array}$.

With regard to shape factor, asymmetry between upper and lower waveform is one of the most characteristic features for tremor acceleration data. The third momentum was commonly used to quantify such a feature (Timmer et al., 1993; Jang et al., 2013). However, the third momentum considers time series data samples in an isolated manner and thus leads to the loss of information in time dimension. To solve the problem, we proposed another feature termed upper-lower symmetry (ULS) index inspired by cross-correlation function (Equation 4). The ULS index evaluates the symmetry of a zero-mean sequence by computing the maximum cross-correlation between its upper and lower waveform (Equations 5, 6). Its value ranges between 0 and 1 and is more sensitive in shape factor than the third momentum.


(4) $$\begin{array}{l} \text{ULS}=\max\;R_{X_{U}X_{L}}\left(\tau \right)=\max\;\overset{\infty }{\underset{n=-\infty }{\sum }}x_{U}^{*}\left(i\right)x_{L}\left(i+\tau \right) \end{array}$$

(5) $$x_{U}(i)=\{\begin{array}{cc} x(i) & \text{sign}(x(i))\geq 0\\ 0 & \text{otherwise} \end{array}$$

(6) $$x_{L}(i)=\{\begin{array}{cc} -x(i) & \text{sign}(x(i))\text{ < }0\\ 0 & \text{otherwise} \end{array}$$

where *x*_*U*_(*i*) denotes the upper waveform sequence, *x*_*L*_(*i*) denotes the lower waveform sequence and *R*_*X*_*U*_*X*_*L*__(τ) denotes the cross-correlation function between the upper and lower waveform with delay τ.

For EMG signal, we computed its approximate entropy (ApEn) with parameters: embedded dimension *m* = 2 and tolerance *r* = 0.2*std. ApEn assessed the shape of the EMG signals by evaluating the regularity and complexity in time domain (Pincus, 1994).

To compare the difference in tremor before and after a certain intervention, we considered two indexes which were: absolute post/pre ratio (Equation 7) and relative post/pre ratio (Equation 8). The absolute post/pre ratio is a basic measure considering mainly the time factor and represents the difference between post-intervention condition and pre-intervention condition. The relative post/pre ratio is a more discreet measure that considers potential confounding factors, such as placebo effect. It was basically calculated as the absolute post/pre ratio of an active tDCS setup subtracted by the absolute post/pre ratio of sham setup. It represented the real difference induced by a certain tDCS setup. Both indexes ranged above 0. A larger post/pre ratio above 1 indicated a more prominent effect of tDCS. On the opposite, a smaller post/pre ratios below 1 indicated a more prominent effect.


(7) $$\begin{array}{l} \text{Absolute post/pre ratio}=\frac{Post_{cur}}{Pre_{cur}} \end{array}$$

where *Post*_*cur*_ and *Pre*_*cur*_ denote the value of an index after and before intervention, respectively.


(8) $$\begin{array}{l} \text{Relative post/pre ratio}=\frac{Post_{active}}{Pre_{active}}-\frac{Post_{sham}}{Pre_{sham}}+1 \end{array}$$

where *Post*_*active*_ and *Pre*_*active*_ denote the value of an index after and before a certain active tDCS intervention, and *Post*_*sham*_ and *Pre*_*sham*_ denote the value of an index after and before sham tDCS.

### 2.7. Statistics

The post/pre ratios of each feature was grouped based on the factor of session (tDCS setup). For absolute post/pre ratios, the data was grouped with four levels (s-tDCS, a-tDCS, c-tDCS, and b-tDCS) while that of the relative post/pre ratios was grouped with three levels (a-tDCS, c-tDCS, and b-tDCS). The Gaussianity of each group and their homogeneity of variance were tested by the Lilliefors test and the Bartlett test. If the null hypothesis of both tests held, namely the groups were both Gaussian and homogeneous in variance, a one-way analysis of variance (ANOVA) with repeated measures was performed, followed by the Tukey-Kramer *post-hoc* analysis. Otherwise, a non-parametric statistical test called the Friedman test was performed, followed by the Nemenyi *post-hoc* analysis. Since the purpose of the analysis over the absolute post/pre ratios was to investigate the effectiveness of the active setups, we considered the performance in the control (s-tDCS) session as a baseline and compared only the pairs between the control (s-tDCS) session and the active tDCS (a-tDCS, c-tDCS, and b-tDCS) sessions in *post-hoc*. In contrast, the relative post/pre ratio was analyzed to investigate the difference between different active tDCS sessions without the baseline effect of the control (s-tDCS) setup. We considered all possible session pairs that were between different active tDCS setups in *post-hoc*.

The baseline stability assumption was verified from two aspects. First, for short-term baseline stability (within one single session), we inspected the pre- and the post-data of each subject and grouped them based on the factor of time with two levels (pre s-tDCS and post s-tDCS). If the null hypothesis of the Lilliefors test and the Bartlett test held, the paired student's t-test was performed. Otherwise, the Wilcoxon signed rank test was performed. Second, for long-term baseline stability (across different sessions), the pre-intervention data of different tDCS setups was targeted and grouped based on the factor of session with 4 levels (pre s-tDCS, pre a-tDCS, pre c-tDCS, and pre b-tDCS). The grouped data was analyzed with the same statistical procedure as in post/pre ratios.

All statistics were performed using Matlab (R2017a, MathWorks Inc., the USA) with the basic level of statistical significance set at *p* < 0.05.

## 3. Results

### 3.1. Baseline stability

A paired student's t-test was performed over the UPDRS-III sum score of the control (s-tDCS) session to evaluate the short-term baseline stability. The result showed there was no significant difference between the pre- and post-data [*t*_(12)_ = −0.433, *p* = 0.672]. Likewise, no significant difference between the pre- and post-data was found in the result by a Wilcoxon signed rank test over the mFTMTRS sum score (*Z* = 0.264, *p* = 0.792), the PPT sum score (*Z* = −0.786, *p* = 0.432), the RMS value of tremor acceleration (*Z* = −0.594, *p* = 0.552) and the RMS value of EMG signal (*Z* = −1.712, *p* = 0.087), respectively.

For long-term baseline stability, only the pre-intervention data of each session was targeted. The result of the repeated-measure ANOVA showed there was no significant effect of the factor of session in the UPDRS-III sum score [*F*_(3,22)_ = 0.544, *p* = 0.580], the RMS value of the tremor acceleration [*F*_(3,33)_ = 0.512, *p* = 0.565] and the RMS value of the EMG signal [*F*_(3,33)_ = 0.873, *p* = 0.396], respectively. In addition, no significant effect in session was found in the mFTMTRS sum score [$\chi_{\left(3\right)}^{2}=3.813,p=0.282$] and the PPT sum score [$\chi_{\left(3\right)}^{2}=4.861,p=0.182$], as revealed by Friedman test.

### 3.2. CTSA

#### 3.2.1. Tremor acceleration

**Figure 4:**
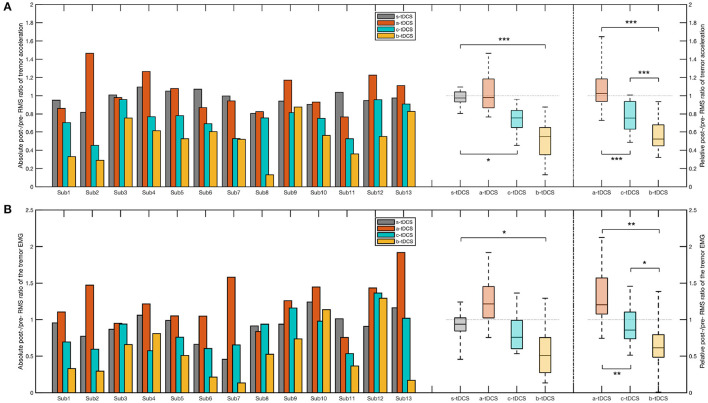
Bar plot and box plot of the post/pre ratios in the CTSA. **(A)** Left: The bar plot and the box plot representing the absolute post-/pre-RMS ratio of the tremor acceleration; Right: The box plot representing the relative post-/pre-RMS ratio of the tremor acceleration. **(B)** Left: The bar plot and the box plot representing the absolute post-/pre-RMS ratio of the EMG signal; Right: The box plot representing the relative post-/pre-RMS ratio of the EMG signal. In the *post-hoc* analysis of the absolute post/pre ratio, we considered only the session pairs between the control (s-tDCS) session and the active tDCS (a-tDCS, c-tDCS, and b-tDCS) session. In the *post-hoc* analysis of the relative post/pre ratios, all session pairs were considered. The significance of *post-hoc* analysis was indicated by asterisks (^*^p < 0.05;^**^ *p* < 0.01;^***^ *p* < 0.001).

For RMS value of tremor acceleration, there was a significant effect of the factor of session in the absolute post/pre ratio [$\chi_{\left(3\right)}^{2}=32.5,p<0.001$] and in the relative post/pre ratio [*F*_(2,22)_ = 12.3, *p* < 0.001]. Compared with the control (s-tDCS) session, we found a significant decrease in the c-tDCS session (*p* = 0.0482) and in the b-tDCS session (*p* < 0.001). The *post-hoc* analysis over the relative post/pre ratio showed the ratio in the b-tDCS was significantly lower than that in the a-tDCS session (*p* < 0.001) and the c-tDCS session (*p* = 0.00635). In addition, the relative post/pre ratio in the c-tDCS session was significantly lower than that in the a-tDCS session (*p* = 0.00311) (Figure 4A).

**Figure 5:**
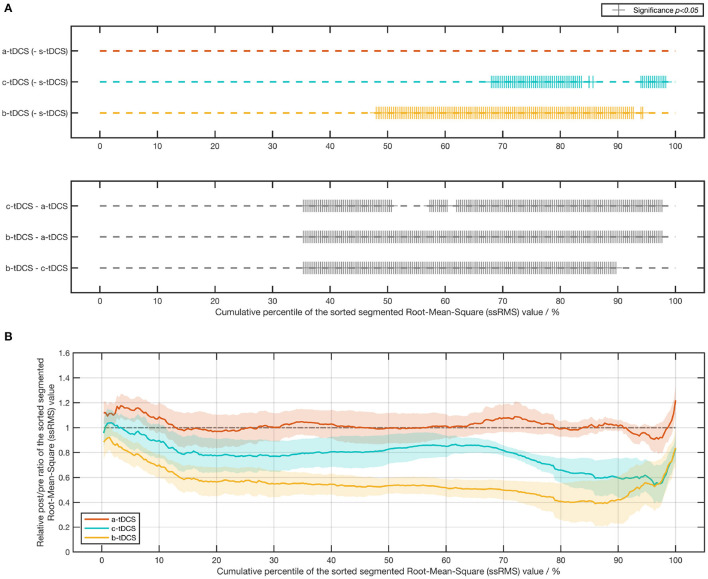
Results of the post/pre ratio of the sorted segmented root-mean-square (ssRMS) values in tremor acceleration. **(A)** Statistical significance of the post-/pre-ssRMS value ratio between different tDCS session pairs. **(B)** Line chart (mean ± SE) of the relative post-/pre-ssRMS value ratio of three active tDCS sessions.

In order to compare in detail how the tremor amplitude differed across various tDCS setups, we computed the absolute post/pre ratio and the relative post/pre ratio of each value in the ssRMS sequence (Figure 5B). The post/pre ratios of the same kind with the same numerical label in the ssRMS sequence were grouped together based on the factor of session and afterwards statistically managed. The outcomes of the significance in the *post-hoc* analysis are shown in Figure 5A, where a plus sign indicates a significant difference between the current session pair at the current percentile. Based on these, the ratio of significant results for each session pair was computed. The b-tDCS session achieved the highest significance ratio of 45.7% among the three concerned active tDCS sessions, compared with the control (s-tDCS) session. The a-tDCS session achieved the lowest significance ratio of 0.00%, while the c-tDCS session achieved a medium significance ratio of 21.3%. When the relative post/pre ratios of active sessions were compared against each other, the significant results of all session pairs were found to occur densely from the 35th percentile to the 97th percentile (correspondingly, from Label No. 106 to Label No. 293 in the ssRMS sequence), as shown in the lower subfigure of Figure 5A. It's worth noting that no significant effect by session was found in either the absolute post/pre ratio [*F*_(3,33)_ = 1.38, *p* = 0.271] or the relative post/pre ratio [*F*_(2,22)_ = 1.50, *p* = 0.247] of the maximum ssRMS value.

With regard to the dominant frequency of bispectrum, no significant effect by session was found in the statistical analysis over the absolute post/pre ratio [$\chi_{\left(3\right)}^{2}=5.21,p=0.157$] and the absolute post/pre ratio [$\chi_{\left(2\right)}^{2}=4.15,p=0.125$], respectively. Likewise, for the ULS index, there was no significant effect by session found in the absolute post/pre ratio [$\chi_{\left(3\right)}^{2}=5.95,p=0.114$] and the absolute post/pre ratio [*F*_(2,22)_ = 0.577, *p* = 0.564], respectively.

#### 3.2.2. EMG signal

In RMS value of EMG signals, we found a significant effect by session in both the absolute post/pre ratio [*F*_(3,33)_ = 3.51, *p* = 0.0466] and the relative post/pre ratio [*F*_(2,22)_ = 4.31, *p* = 0.0452], respectively. In the *post-hoc* analysis over the absolute post/pre ratio, a significant difference between the b-tDCS session and the control (s-tDCS) session was found (*p* = 0.0113). There was no significant difference between the a-tDCS session and the control (s-tDCS) session (*p* = 0.686), and between the c-tDCS session and the control (s-tDCS) session (*p* = 0.0576). In comparing between different active tDCS sessions, the *post-hoc* analysis over the relative post/pre ratio revealed both the c-tDCS session (*p* = 0.00521) and the b-tDCS (*p* = 0.00152) has a lower relative post/pre ratio than the a-tDCS session. In addition, the b-tDCS session yielded the lowest relative post/pre ratio, even compared with the c-tDCS session (*p* = 0.0136) (Figure 4B).

No significant effect by session was found in the absolute post/pre ratio and the relative post/pre ratio of ZC [$\chi_{\left(3\right)}^{2}=3.92,p=0.270$]. Likewise, there was no significant session effect in the absolute post/pre ratio and the relative post/pre ratio of ApEn [$\chi^{2}(3)=5.77,p=0.123$].

### 3.3. Clinical scales

#### 3.3.1. UPDRS-III

**Figure 6:**
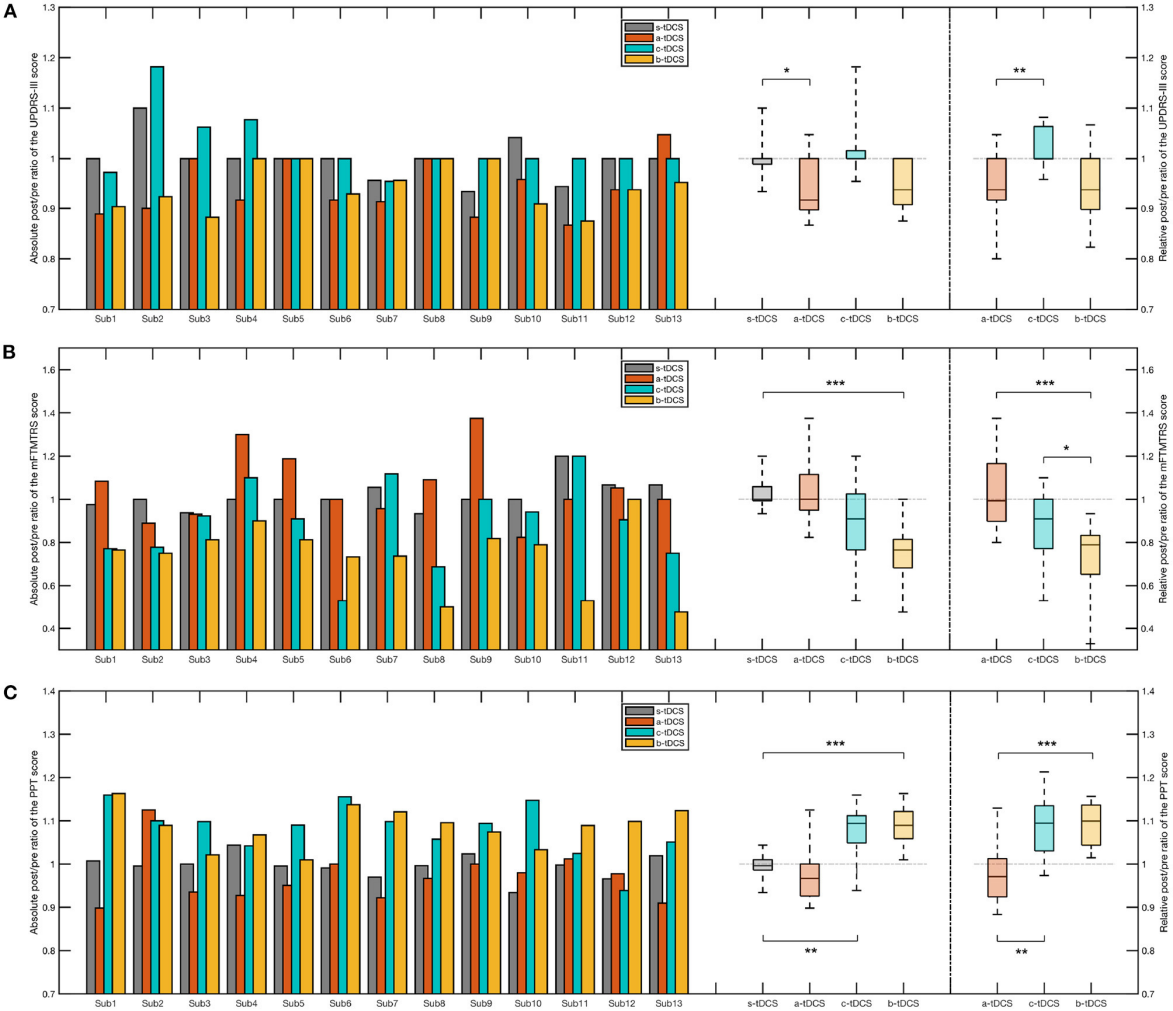
Bar plot and box plot of the post/pre ratios in different clinical measures. **(A)** Left: The bar plot and the box plot representing the absolute post/pre ratio of the UPDRS-III sum score; Right: The box plot representing the relative post/pre ratio of the UPDRS-III sum score. **(B)** Left: The bar plot and the box plot representing the absolute post/pre ratio of the mFTMTRS sum score; Right: The box plot representing the relative post/pre ratio of the mFTMTRS sum score. **(C)** Left: The bar plot and the box plot representing the absolute post/pre ratio of the PPT sum score; Right: The box plot representing the relative post/pre ratio of the PPT sum score. In the *post-hoc* analysis of the absolute post/pre ratio, we considered only the session pairs between the control (s-tDCS) session and the active tDCS (a-tDCS, c-tDCS, and b-tDCS) session. In the *post-hoc* analysis of the relative post/pre ratios, all session pairs were considered. The significance of *post-hoc* analysis was indicated by asterisks (^*^p < 0.05; ^**^p < 0.01; ^***^p < 0.001).

To investigate the effectiveness of the three concerned tDCS setups, a Friedman test was performed over the absolute post/pre ratio of the sum score. The result showed there was a significant effect of the factor of session [$\chi_{\left(3\right)}^{2}=16.1$, *p* = 0.00110]. Post-hoc analysis revealed, among the three concerned active setups, only the a-tDCS session resulted in a significant reduction of the absolute post/pre ratio compared with the control (s-tDCS) session (*p* = 0.0186). Likewise, we found a significant effect of session in the relative post/pre ratio [$\chi_{\left(3\right)}^{2}=11.3,p=0.00354$] and that the relative post/pre ratio in the a-tDCS session was significantly lower than that in the c-tDCS session in *post-hoc* (*p* = 0.0031) (Figure 6A).

When we only considered the items related to tremor, namely item 20 and item 21 of UPDRS-III (UPDRS-III-tr), a significant reduction of the absolute post/pre ratio was found in the b-tDCS session compared to the control (s-tDCS) session (*p* < 0.001). There was no significant difference between the a-tDCS session and the control (s-tDCS) session (*p* = 1.00) and no significant difference between the c-tDCS session and the control (s-tDCS) session (*p* = 0.606). There was a significant effect of the session factor in the relative post/pre ratio [χ^2^(3) = 24.3, *p* < 0.001]. *Post-hoc* analysis indicated there was a significant decrease in the b-tDCS session compared with the a-tDCS session (*p* < 0.001) and the c-tDCS session (*p* = 0.0164), respectively.

#### 3.3.2. mFTMTRS

Considering the sum score of mFTMTRS, there was a significant effect of the factor of session over the absolute post/pre ratio [$\chi_{\left(3\right)}^{2}=24.3,p<0.001$]. Among the three active tDCS sessions, only the b-tDCS session generated a significant difference compared with the control (s-tDCS) session (*p* < 0.001). With regard to the relative post/pre ratio, a significant increase was found in the b-tDCS session, compared with the a-tDCS session (*p* < 0.001) and the c-tDCS session (*p* = 0.0488), respectively (Figure 6B).

In analyzing the sum score of mFTMTRS-MAS, we found a significant effect by the factor of session in the absolute post/pre-ratio [$\chi_{\left(3\right)}^{2}=18.4,p<0.001$] and a significant decrease in the b-tDCS session compared with the control (s-tDCS) session (*p* < 0.001). The result of the *post-hoc* analysis over the relative post/pre ratio showed there was a significant decrease in the b-tDCS session compared with the a-tDCS session (*p* = 0.0035). Similar results were found in the analysis over the sum score of mFTMTRS-LAS. In an attempt to investigate whether there was a difference in the effect of tDCS between MAS and LAS, we computed the difference of the relative post/pre ratio between the mFTMTRS-MAS sum score and the mFTMTRS-LAS. A Friedman test was performed over the computed difference. However, no significant effect of session was found [$\chi_{\left(2\right)}^{2}=0.0426,p=0.979$].

#### 3.3.3. PPT

A repeated-measure ANOVA was performed over the absolute post/pre ratio of the PPT sum score, where a significant effect of session was found [*F*_(3,33)_ = 6.28, *p* = 0.00323]. The Tukey-Kramer *post-hoc* analysis showed there was a significant increase respectively in the c-tDCS session (*p* = 0.00339) and in the b-tDCS session (*p* < 0.001), compared with the s-tDCS session. The *post-hoc* analysis over the relative post/pre ratio revealed a significant difference between the c-tDCS session and the a-tDCS session (*p* = 0.00541), and between the b-tDCS session and the a-tDCS session (*p* < 0.001), respectively (Figure 6C).

Considering the sum score of PPT-MAS, we found a significant effect of the session factor in the absolute post/pre ratio [$\chi_{\left(3\right)}^{2}=27.5,p<0.001$] and the relative post/pre ratio [$\chi_{\left(3\right)}^{2}=17.2,p<0.001$], respectively. The *post hoc* analysis over the absolute post/pre ratio showed there was a significant increase in the c-tDCS session (*p* = 0.0096) and the b-tDCS session (*p* < 0.001) respectively, compared with the control (s-tDCS) session. A significant increase in the c-tDCS session (*p* = 0.0048) and in the b-tDCS session (*p* < 0.001), compared with the a-tDCS session, was found in statistical analysis over the relative post/pre ratio. With regard to the sum score of PPT-LAS, the statistical analysis revealed a result that was similar to that of PPT-MAS. To investigate whether there was a difference between MAS and LAS in the effect of active tDCS setups, a repeated-measure ANOVA was performed over the relative post/pre ratio difference between PPT-MAS and PPT-LAS. However, no significant effect of session was found [*F*_(2,22)_ = 2.14, *p* = 0.147].

### 3.4. Correlation between different measures

**Figure 7:**
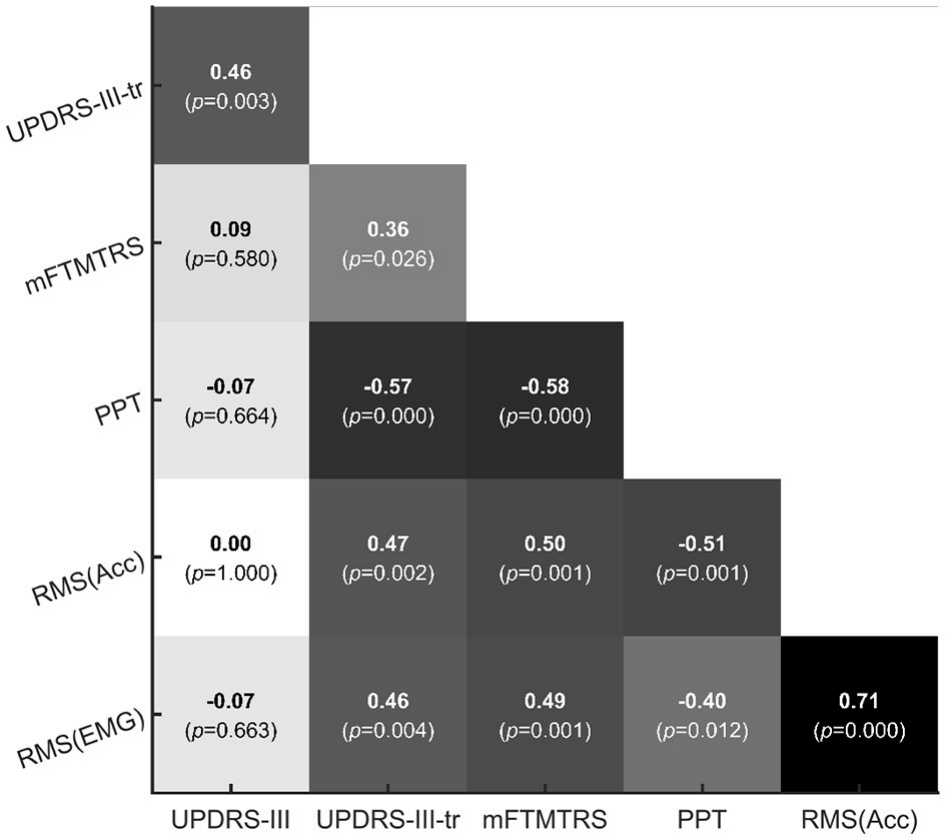
Correlation matrix of the relative post/pre ratio in different measures.

To investigate the correlation between different measures, a Spearman correlation analysis was performed over the relative post/pre ratio of the UPDRS-III sum score, the UPDRS-III-tr sum score, the mFTMTRS sum score, the PPT sum score, the RMS value of tremor acceleration and the RMS value of the EMG signal. The result of the Spearman correlation matrix is shown in Figure 7, where a darker background color indicates a higher Spearman correlation coefficient, and vice versa. The highest Spearman coefficient was found between the RMS value of tremor acceleration and the RMS value of EMG signal (ρ = 0.71, *p* < 0.001), followed by the Spearman coefficient between the PPT sum score and the mFTMTRS sum score (ρ = −0.58, *p* < 0.001). The UPDRS-III sum score had the lowest correlation coefficients with the other measures. However, the UPDRS-III-tr sum score had a much higher correlation coefficient with the other measures than the UPDRS-III sum score did.

## 4. Conclusion and discussion

In the research, we designed a randomized, sham-controlled, double-blind, crossover experiment to investigate the immediate response of Parkinsonian tremor with different tDCS setups. In addition to the control (s-tDCS) setup, there were three active tDCS setups, namely the a-tDCS setup, the c-tDCS setup and the b-tDCS setup, with each corresponding to one of the randomized sessions in the experiment. In each session, our subjects underwent a similar set of evaluation procedure before and after the intervention. The effect of one specific tDCS setup was evaluated by comparing the pre-intervention and post-intervention performance of the subjects. We investigated the effectiveness of a specific active tDCS setup by comparing it with the sham tDCS setup in the absolute post/pre ratio while the difference between different active tDCS setups were evaluated by comparing their relative post/pre ratio where the effect resulted from the sham setup were removed as a baseline.

### 4.1. Effectiveness of different tDCS setups

The baseline stability assumption was verified by inspecting carefully whether subjects could maintain a constant motor performance with different measures in both the control session and before intervention across different sessions. Consistent with the results of the study by Fregni et al. (2006b), we found only the a-tDCS setup resulted in a significant reduction in the sum score of the UPDRS-III compared with the control (s-tDCS) setup, indicating that only the a-tDCS had a positive effect over the motor function related to Parkinson's disease. However, when we looked into the subscale related to tremor severity (item 20 and item 21 of UPDRS-III), our results showed only the b-tDCS setup resulted in a significant reduction of the sum score and outperformed the other active tDCS setups in suppressing tremor, which was contrary to the result of UPDRS-III sum score. In the mFTMTRS assessment, only the b-tDCS setup brought a significant reduction in the sum score of the mFTMTRS compared with the control (s-tDCS), indicating the b-tDCS setup was effective in suppressing the intensity of tremor. The results of the PPT assessment showed the c-tDCS setup and the b-tDCS setup were both effective in improving hand manipulative dexterity, indicating a potential improvement in the intensity of hand tremor by c-tdCS and b-tDCS. In addition, no significant effect was found in the a-tDCS setup. Differently, a study by Fregni et al. (2006b) showed no significant effect of either a-tDCS or c-tDCS with the measure of PPT. We considered several possible contributing factors: first, the number of subjects [*N*(a-tDCS) = 9, *N*(c-tDCS) = 8] in their study was smaller than that in ours (*N* = 13), which might cause the lack of power in statistics. Second, the current intensity and density transduced through the sponge electrodes in their experiment [*I*(tDCS) = 1.0mA, *J*(tDCS) = 0.0286mA/cm^2^] were smaller than those in our experiment [*I*(tDCS) = 1.5 mA, *J*(*tDCS*)= 0.0355mA/cm^2^], indicating that our setup might induce stronger effect by reaching the deeper structure of the brain and thus produced a more significant result. In terms of the CTSA, the c-tDCS setup and the b-tDCS setup both resulted in a significant reduction in the intensity of tremor acceleration. In addition, the optimal effect of tremor suppression was obtained under the bilateral setup. For EMG signal, the statistical results showed only the b-tDCS setup among the three concerned active tDCS setups was effective in reducing tremor-related muscle activities, namely, the b-tDCS setup could suppress tremor more effectively than the other tDCS setups. No significant effect of tDCS with regard to tremor frequency and shape was found in either the tremor acceleration or the EMG signal.

Taken together, our results revealed the technique of tDCS was effective in suppressing tremor and that although a-tDCS benefited Parkinson's disease more as revealed by UPDRS-III, the b-tDCS and c-tDCS setup might be more beneficial when targeting the symptom of Parkinsonian tremor solely. Among the three active tDCS setups, the b-tDCS setup outperformed the others in suppressing tremor. Therefore, in treating Parkinsonian tremor, we proposed the use of the c-tDCS or b-tDCS setup, especially the b-tDCS setup, rather than the a-tDCS setup that was widely accepted in tDCS treatment over Parkinson's disease in previous studies. In addition, our research suggested different symptoms of Parkinson's disease might require different tDCS setups to induce an optimal treatment effect.

### 4.2. Crossover design of the experiment

As reported by previous studies, the effect of tDCS over human bodies may be unstable or even contradictive in similar experimental setups (López-Alonso et al., 2014, 2015). We attributed these discrepancies to the mental factors, such as the placebo effect, which originated mentally from the experiment and affected the performance of subjects physically in return. Under consideration of such factors, our experiment followed a crossover design. The aim was to rule out the subject-specific factors by taking the baseline performance of the subjects into consideration. We assessed the net effect of a specific active tDCS setup by first computing its absolute post/pre ratio and then detracting the individual-specific baseline post/pre ratio of the control (s-tDCS). We assumed that the crossover design increased the reliability of our study. The results of the statistical analysis demonstrated our assumption and that the crossover design was indispensable in exploring the explicit effect of tDCS.

One of the disadvantages resulted from a crossover design was its additional physical and mental burden over subjects. To alleviate the problem, we suggested a long enough time interval of over 48 hours between intervention sessions. The feedback from our subjects showed all subjects tolerated the experiment burden well. Another potential problem resulted from a crossover design was the familiarization effect, with which subjects might perform better in the experiment as time elapsed. The involvement of familiarization effect might lead in type-2 error in our statistics. To check whether the familiarization effect is significant, we re-grouped our data with the factor of stimulation order and performed statistical analysis. Results showed no significant effect of stimulation order in UPDRS-III sum score [$\chi_{\left(3\right)}^{2}=0.735,p=0.865$], mFTMTRS sum score [*F*_(3,33)_ = 0.835, *p* = 0.466], PPT sum score [*F*_(3,33)_ = 2.22, *p* = 0.131], RMS value of tremor acceleration [*F*_(3,33)_ = 1.68, *p* = 0.210] and RMS value of EMG signal [*F*_(3,33)_ = 1.01, *p* = 0.387], respectively. Therefore, we concluded the familiarization effect didn't significantly affect our result in our experiment.

### 4.3. Association between different measures

To assess tremor, three different clinical measures and a self-design evaluation related to continuous tremor signals were utilized in the experiment. Among the four measures utilized in the experiment, the CTSA along with its subsequent analysis was theoretically more objective and less operator-dependent since it required only a series of standardized procedure. It could additionally provide more detailed information in depicting tremor. Based on previous studies, the traditional clinical scales that require a specific physician to score each item with a few levels (such as UPDRS, each item of which has five levels: “Normal,” “Slight,” “Mild,” “Moderate” and “Severe”) may be more operator-dependent and less specific in setting (Palmer et al., 2010). Therefore, in this research, we computed the correlation between the CTSA and the other measures to provide a feedback to evaluate the objectiveness of the other chosen clinical measures. Among the results of the Spearman correlation analysis, the most correlated result was within the CTSA, namely between the RMS value of the tremor acceleration and the RMS value of the EMG signal (ρ = 0.71, *p* < 0.001). The correlation between the CTSA and the other measures was relatively lower and reached a coefficient of approximately 0.50 with significance, indicating the potential insufficiency of traditional clinical measures in both accuracy and sensitivity while measuring tremor. The correlation coefficient between the three clinical measures reached up to 0.58, which was slightly lower than that within the CTSA. Specifically, between the UPDRS-III sum score and other measures, the correlation coefficient was as low as almost zero, probably because the UPDRS-III considers symptoms more than tremor. Our results suggested that traditional clinical scales might be potentially insufficient in depicting especially the instant response of tremor. The use of less-operator-dependent and more-objective tools, such as CTSA, was more recommended in measuring tremor.

### 4.4. Mechanism underlying tDCS in suppressing Parkinsonian tremor

As two of the most fundamental electrode setups of tDCS, the anodal setup and the cathodal setup were included in the experimental protocol. In general, the effect of tDCS over primary cortex is polarity-dependent, namely, a-tDCS facilitates while c-tDCS inhibits contralateral motor cortex excitability (Nitsche and Paulus, 2000, 2001). However, based on our findings, tremor was found to decrease more in intensity after the c-tDCS session. We considered two possibilities that might explain this:

First, our result may be directly linked to the excitability alteration of sensorimotor cortex which plays a positive correlative modulatory role in tremorgenesis. The study by Fregni et al. (2006b) reported substantial evidence of c-tDCS in lowering PD subjects' motor cortex excitability with MEP assessment. Thus, the suppressed motor cortex with c-tDCS may result in a lower tremor intensity. In contrast, the facilitating effect of a-tDCS in motor cortex excitability may accordingly lead to an uplift in tremor intensity. However, within our results, no significant increase of tremor was found with a-tDCS. We assumed the intensified components of tremor might be offset by the beneficial effect of the increased dopamine concentration induced by a-tDCS (Fonteneau et al., 2018). Therefore, a-tDCS might exert a beneficial and adverse effect over tremor simultaneously. This was in line with the findings by Fregni et al. (2006b), who found the improvement of tremor after a-tDCS was much less than that in either rigidity or bradykinesia.

Second, the function of c-tDCS might be related to the disruption hypothesis of DBS, where abnormal information flow was disrupted. Although, based on the results of simulated models, the stimuli of tDCS might not reach the depth as DBS did (Dannhauer et al., 2012), tDCS might function by involving in the complex transmitter modulation through the motor cortex to disconnect the basal ganglia-thalamo-cortical loop (Jitkritsadakul et al., 2015). Within our results of the ssRMS sequence, we found after c-tDCS there was no significant change in the maximum ssRMS value of tremor acceleration, regardless of the significant reduction in the average RMS value. This was similar to the effect of DBS in tremor, which could be best described as altering the “on/off” state of tremor, that is, increasing the time of the “on” state while reducing that of the “off” state (Hawley et al., 2014), rather than, reducing tremor intensity directly. Substantial evidence has been reported in, such as, a previous study by Beuter and Titcombe (2003), who found little effect of DBS with low-amplitude resting tremor. In addition, another long-term study showed clearly that DBS increased the “on” state time in the day approximately from originally 25% to 75%, that is, for about 25% of the day, patients remained at the “off” state with DBS. With regard to how c-tDCS affected tremor, a most recent study by Bachtiar et al. (2018) investigated the setup of c-tDCS over the more-affected M1 and found a reduction of GABA concentration in the less-affected M1. A recent study by van Nuland et al. (2020) revealed the GABA concentration was negatively correlated with the tremor severity. Thus, the setup of c-tDCS led to a reduction of tremor severity.

The setup of b-tDCS might have a different mechanism to interact with tremor. Some relevant evidence has been reported: first, with b-tDCS, the cortical currents were found to transduce in a more lateral to medial orientation, compared to the setup of either a-tDCS or c-tDCS, where the currents were transduced in a more dorsal to ventral direction (Wagner et al., 2007). The difference in current flows could lead to the activation in different cerebral structure that contributed to different pathways of tremor suppression. Second, Bachtiar et al. (2018) have reported a reduction of GABA concentration in the more-affected M1 after b-tDCS, which was different from that after c-tDCS where the GABA concentration reduction was found in the contralateral M1. Besides, a study comparing b-tDCS and a-tDCS found an ongoing increase in intracortical functional connectivity only after b-tDCS rather than a-tDCS (Sehm et al., 2013). Another study by Mordillo-Mateos et al. (2012) assessed the cortical excitability using motor-evoked potential and proved b-tDCS could induced a combined effect of a-tDCS and c-tDCS in shifting cortical excitability. A clinical study related to stroke combined with b-tDCS as well as unilateral tDCS showed the effect b-tDCS outperformed that of either a-tDCS or c-tDCS, and proposed b-tDCS for rectifying interhemispheric imbalances in stroke (Mahmoudi et al., 2011). This might also be the case in Parkinsonian tremor, whose onset and progress in general are unilateral and unbalanced. Additionally, according to our results, no bilateral difference (difference between MAS and LAS) of the effect of tDCS was found in terms of FTMTRS and PPT, respectively. Thus, our results in return might reveal an underlying interhemispheric unbalance of cortical excitability induced by Parkinsonian tremor, which could be rectified with tDCS. However, to further prove this, more substantial evidence and research are needed.

### 4.5. Highlights, limitations and future work

To the best of our knowledge, this is the first randomized, sham-controlled, double-blind, crossover research that compared the immediate effect of different tDCS setups targeting Parkinsonian tremor solely. In addition, this is the first time the bilateral tDCS setup, with the cathode over the more affected M1 and the anode over the contralateral M1, was introduced to the treatment of Parkinson's disease. A self-design assessment termed the CTSA was specifically designed for a more accurate and detailed evaluation of the continuous tremor signals. A few novel features, such as the ULS index and the ssRMS sequence to quantify tremor shape and sub-regional tremor amplitude, and the index of the significance ratio for comparison between session pairs were proposed in the paper. With regard to the limitation of the research, a limited number of subjects participated in the experiment because of the time and effort cost due to the crossover design. More cases of qualified subjects are needed to fully verify the efficiency of different tDCS setups in the future. Another limitation of the experiment is the lack of the involvement of cerebral monitoring techniques, such as electroencephalography (EEG) or functional magnetic resonance imaging (fMRI), which may provide information on cortical excitability alteration and help reveal the mechanism of tDCS better. In the future, we may systematically investigate both the short-term and long-term effect of different bilateral tDCS setups over Parkinson's disease as a continuation of the current research.

## Data availability statement

The raw data supporting the conclusions of this article will be made available by the authors, without undue reservation.

## Ethics statement

The studies involving human participants were reviewed and approved by Ethics Committee of Shanghai Jiao Tong University. The patients/participants provided their written informed consent to participate in this study.

## Author contributions

BZ contributed in carrying out the experiment, data analysis, and drafting the manuscript. FH contributed in carrying out the experiment and coordinating the process of patient recruitment and participation. JL contributed in coordinating the process of patient recruitment and participation. DZ conceived of the study and gave instructions on experimental design. All authors contributed to the article and approved the submitted version.

## References

[B1] Bachtiar V. Johnstone A. Berrington A. Lemke C. Johansen-Berg H. Emir U.. (2018). Modulating regional motor cortical excitability with noninvasive brain stimulation results in neurochemical changes in bilateral motor cortices. J. Neurosci. 38, 7327–7336. 10.1523/JNEUROSCI.2853-17.2018 30030397 PMC6096041

[B2] Benabid A. L. (2003). Deep brain stimulation for Parkinson's disease. Curr. Opin. Neurobiol. 13, 696–706. 10.1016/j.conb.2003.11.001 14662371

[B3] Benninger D. H. Lomarev M. Lopez G. Wassermann E. M. Li X. Considine E.. (2010). Transcranial direct current stimulation for the treatment of Parkinson's disease. J. Neurol. Neurosurg. Psychiatry 81, 1105–1111. 10.1136/jnnp.2009.202556 20870863 PMC4162743

[B4] Berk C. Carr J. Sinden M. Martzke J. Honey C. R. (2002). Thalamic deep brain stimulation for the treatment of tremor due to multiple sclerosis: a prospective study of tremor and quality of life. J. Neurosurg. 97, 815–820. 10.3171/jns.2002.97.4.0815 12405368

[B5] Beuter A. Titcombe M. S. (2003). Modulation of tremor amplitude during deep brain stimulation at different frequencies. Brain Cogn. 53, 190–192. 10.1016/S0278-2626(03)00107-6 14607145

[B6] Bikson M. Grossman P. Thomas C. Zannou A. L. Jiang J. Adnan T.. (2016). Safety of transcranial direct current stimulation: evidence based update 2016. Brain Stimul. 9, 641–661. 10.1016/j.brs.2016.06.004 27372845 PMC5007190

[B7] Boggio P. S. Ferrucci R. Rigonatti S. P. Covre P. Nitsche M. Pascual-Leone A.. (2006). Effects of transcranial direct current stimulation on working memory in patients with Parkinson's disease. J. Neurol. Sci. 249, 31–38. 10.1016/j.jns.2006.05.062 16843494

[B8] Borovac J. A. (2016). Side effects of a dopamine agonist therapy for Parkinson's disease: a mini-review of clinical pharmacology. Yale J. Biol. Med. 89, 37–47. 27505015 PMC4797835

[B9] Bronstein J. M. Tagliati M. Alterman R. L. Lozano A. M. Volkmann J. Stefani A.. (2011). Deep brain stimulation for Parkinson disease: an expert consensus and review of key issues. Arch. Neurol. 68, 165–165. 10.1001/archneurol.2010.260 20937936 PMC4523130

[B10] Coffman B. A. Clark V. P. Parasuraman R. (2014). Battery powered thought: enhancement of attention, learning, and memory in healthy adults using transcranial direct current stimulation. Neuroimage 85, 895–908. 10.1016/j.neuroimage.2013.07.083 23933040

[B11] Costa-Ribeiro A. Maux A. Bosford T. Aoki Y. Castro R. Baltar A.. (2017). Transcranial direct current stimulation associated with gait training in Parkinson's disease: a pilot randomized clinical trial. Develop. Neurorehabil. 20, 121–128. 10.3109/17518423.2015.1131755 26864140

[B12] Dannhauer M. Brooks D. Tucker D. MacLeod R. (2012). “A pipeline for the simulation of transcranial direct current stimulation for realistic human head models using SCIRUN/BIOMESH3D,” in 2012 Annual International Conference of the IEEE Engineering in Medicine and Biology Society. IEEE, 5486–5489. 10.1109/EMBC.2012.6347236 23367171 PMC3651514

[B13] Deuschl G. Bain P. Brin M. Committee A. H. S. (1998). Consensus statement of the movement disorder society on tremor. Mov. Disord. 13, 2–23. 10.1002/mds.870131303 9827589

[B14] Di Lazzaro V. Dileone M. Capone F. Pellegrino G. Ranieri F. Musumeci G.. (2014). Immediate and late modulation of interhemipheric imbalance with bilateral transcranial direct current stimulation in acute stroke. Brain Stimul. 7, 841–848. 10.1016/j.brs.2014.10.001 25458712

[B15] Diamond A. Jankovic J. (2005). The effect of deep brain stimulation on quality of life in movement disorders. J. Neurol. Neurosurg. Psychiatry 76, 1188–1193. 10.1136/jnnp.2005.065334 16107348 PMC1739801

[B16] Dick F. Semple S. Chen R. Seaton A. (2000). Neurological deficits in solvent-exposed painters: a syndrome including impaired colour vision, cognitive defects, tremor and loss of vibration sensation. QJM 93, 655–661. 10.1093/qjmed/93.10.655 11029475

[B17] Doruk D. Gray Z. Bravo G. L. Pascual-Leone A. Fregni F. (2014). Effects of tdcs on executive function in Parkinson's disease. Neurosci. Lett. 582, 27–31. 10.1016/j.neulet.2014.08.043 25179996

[B18] Doshi P. K. (2011). Long-term surgical and hardware-related complications of deep brain stimulation. Stereotact. Funct. Neuros. 89, 89–95. 10.1159/000323372 21293168

[B19] Fahn S. Tolosa E. Marín C. (1993). Clinical rating scale for tremor. Parkinson's Dis. Mov. Disord. 2, 271–280.

[B20] Fernández-Lago H. Bello O. Mora-Cerdá F. Montero-Cámara J. Fernández-del Olmo M. Á. (2017). Treadmill walking combined with anodal transcranial direct current stimulation in Parkinson disease: a pilot study of kinematic and neurophysiological effects. Am. J. Phys. Med. Rehabil. 96, 801–808. 10.1097/PHM.0000000000000751 28398968

[B21] Fonteneau C. Redoute J. Haesebaert F. Le Bars D. Costes N. Suaud-Chagny M.-F.. (2018). Frontal transcranial direct current stimulation induces dopamine release in the ventral striatum in human. Cereb. Cortex 28, 2636–2646. 10.1093/cercor/bhy093 29688276 PMC5998959

[B22] Fregni F. Boggio P. S. Mansur C. G. Wagner T. Ferreira M. J. Lima M. C.. (2005). Transcranial direct current stimulation of the unaffected hemisphere in stroke patients. Neuroreport 16, 1551–1555. 10.1097/01.wnr.0000177010.44602.5e 16148743

[B23] Fregni F. Boggio P. S. Nitsche M. A. Marcolin M. A. Rigonatti S. P. Pascual-Leone A. (2006a). Treatment of major depression with transcranial direct current stimulation. Bipolar Disord. 8, 203–204. 10.1111/j.1399-5618.2006.00291.x 16542193

[B24] Fregni F. Boggio P. S. Santos M. C. Lima M. Vieira A. L. Rigonatti S. P.. (2006b). Noninvasive cortical stimulation with transcranial direct current stimulation in Parkinson's disease. Mov. Disord. 21, 1693–1702. 10.1002/mds.21012 16817194

[B25] Goetz C. G. Tilley B. C. Shaftman S. R. Stebbins G. T. Fahn S. Martinez-Martin P.. (2008). Movement disorder society-sponsored revision of the unified Parkinson's disease rating scale (MDS-UPDRS): scale presentation and clinimetric testing results. Movement Disord. 23, 2129–2170. 10.1002/mds.22340 19025984

[B26] Gray P. Hildebrand K. (2000). Fall risk factors in Parkinson's disease. J. Neurosci. Nurs. 32, 222. 10.1097/01376517-200008000-00006 10994536

[B27] Hadoush H. Al-Jarrah M. Khalil H. Al-Sharman A. Al-Ghazawi S. (2018). Bilateral anodal transcranial direct current stimulation effect on balance and fearing of fall in patient with Parkinson's disease. NeuroRehabilitation 42, 63–68. 10.3233/NRE-172212 29400676

[B28] Hallett M. (2007). Transcranial magnetic stimulation: a primer. Neuron 55, 187–199. 10.1016/j.neuron.2007.06.026 17640522

[B29] Hawley J. S. Armstrong M. J. Weiner W. J. (2014). Parkinson's Disease: Improving Patient Care. Oxford: Oxford University Press.

[B30] Helmich R. C. Toni I. Deuschl G. Bloem B. R. (2013). The pathophysiology of essential tremor and Parkinson's tremor. Curr. Neurol. Neurosci. Rep. 13, 378. 10.1007/s11910-013-0378-8 23893097

[B31] Hoehn M. M. Yahr M. D. (1967). Parkinsonism: onset, progression, and mortality. Neurology 17, 427–427. 10.1212/WNL.17.5.427 6067254

[B32] Jang W. Han J. Park J. Kim J. Cho J. Koh S. B.. (2013). Waveform analysis of tremor may help to differentiate Parkinson's disease from drug-induced Parkinsonism. Physiol. Meas. 34, N15. 10.1088/0967-3334/34/3/N15 23442947

[B33] Jitkritsadakul O. Thanawattano C. Anan C. Bhidayasiri R. (2015). Exploring the effect of electrical muscle stimulation as a novel treatment of intractable tremor in Parkinson's disease. J. Neurol. Sci. 358, 146–152. 10.1016/j.jns.2015.08.1527 26342942

[B34] Kaski D. Allum J. Bronstein A. Dominguez R. (2014). Applying anodal tDCS during tango dancing in a patient with Parkinson's disease. Neurosci. Lett. 568, 39–43. 10.1016/j.neulet.2014.03.043 24686184

[B35] Kleiner-Fisman G. Herzog J. Fisman D. N. Tamma F. Lyons K. E. Pahwa R.. (2006). Subthalamic nucleus deep brain stimulation: summary and meta-analysis of outcomes. Mov. Disord. 21, S290–S304. 10.1002/mds.20962 16892449

[B36] Lee A. Furuya S. Altenmüller E. (2014). Epidemiology and treatment of 23 musicians with task specific tremor. J. Clin. Mov. Disord. 1, 5. 10.1186/2054-7072-1-5 26788331 PMC4677731

[B37] Lee D. J. Dallapiazza R. F. De Vloo P. Lozano A. M. (2018). Current surgical treatments for Parkinson's disease and potential therapeutic targets. Neural Regenerat. Res. 13, 1342. 10.4103/1673-5374.235220 30106037 PMC6108190

[B38] López-Alonso V. Cheeran B. Río-Rodríguez D. Fernández-del Olmo M. (2014). Inter-individual variability in response to non-invasive brain stimulation paradigms. Brain Stimul. 7, 372–380. 10.1016/j.brs.2014.02.004 24630849

[B39] López-Alonso V. Fernández-del Olmo M. Costantini A. Gonzalez-Henriquez J. J. Cheeran B. (2015). Intra-individual variability in the response to anodal transcranial direct current stimulation. Clin. Neurophysiol. 126, 2342–2347. 10.1016/j.clinph.2015.03.022 25922127

[B40] Mahmoudi H. Haghighi A. B. Petramfar P. Jahanshahi S. Salehi Z. Fregni F. (2011). Transcranial direct current stimulation: electrode montage in stroke. Disabil. Rehabil. 33, 1383–1388. 10.3109/09638288.2010.532283 21110732

[B41] Manenti R. Brambilla M. Rosini S. Orizio I. Ferrari C. Borroni B.. (2014). Time up and go task performance improves after transcranial direct current stimulation in patient affected by Parkinson's disease. Neurosci. Lett. 580, 74–77. 10.1016/j.neulet.2014.07.052 25107738

[B42] Manenti R. Cotelli M. S. Cobelli C. Gobbi E. Brambilla M. Rusich D.. (2018). Transcranial direct current stimulation combined with cognitive training for the treatment of Parkinson disease: a randomized, placebo-controlled study. Brain Stimul. 11, 1251–1262. 10.1016/j.brs.2018.07.046 30056141

[B43] Maneski L. P. Jorgovanović N. Ilić V. Došen S. Keller T. Popović M. B.. (2011). Electrical stimulation for the suppression of pathological tremor. Med. Biol. Eng. Comput. 49, 1187. 10.1007/s11517-011-0803-6 21755318

[B44] Merletti R. Di Torino P. (1999). Standards for reporting emg data. J. Electromyogr. Kinesiol. 9, 3–4.

[B45] Mordillo-Mateos L. Turpin-Fenoll L. Millán-Pascual J. Nú nez-Pérez N. Panyavin I. Gómez-Argüelles J. M.. (2012). Effects of simultaneous bilateral tDCS of the human motor cortex. Brain Stimul. 5, 214–222. 10.1016/j.brs.2011.05.001 21782545

[B46] Nair D. G. Renga V. Lindenberg R. Zhu L. Schlaug G. (2011). Optimizing recovery potential through simultaneous occupational therapy and non-invasive brain-stimulation using tDCS. Restorat. Neurol. Neurosci. 29, 411–420. 10.3233/RNN-2011-0612 22124031 PMC4425274

[B47] Nitsche M. A. Cohen L. G. Wassermann E. M. Priori A. Lang N. Antal A.. (2008). Transcranial direct current stimulation: state of the art 2008. Brain Stimul. 1, 206–223. 10.1016/j.brs.2008.06.004 20633386

[B48] Nitsche M. A. Paulus W. (2000). Excitability changes induced in the human motor cortex by weak transcranial direct current stimulation. J. Physiol. 527, 633–639. 10.1111/j.1469-7793.2000.t01-1-00633.x 10990547 PMC2270099

[B49] Nitsche M. A. Paulus W. (2001). Sustained excitability elevations induced by transcranial dc motor cortex stimulation in humans. Neurology 57, 1899–1901. 10.1212/WNL.57.10.1899 11723286

[B50] Nonnekes J. Timmer M. H. de Vries N. M. Rascol O. Helmich R. C. Bloem B. R. (2016). Unmasking levodopa resistance in Parkinson's disease. Mov. Disord. 31, 1602–1609. 10.1002/mds.26712 27430479

[B51] Palm U. Reisinger E. Keeser D. Kuo M.-F. Pogarell O. Leicht G.. (2013). Evaluation of sham transcranial direct current stimulation for randomized, placebo-controlled clinical trials. Brain Stimul. 6, 690–695. 10.1016/j.brs.2013.01.005 23415938

[B52] Palmer J. L. Coats M. A. Roe C. M. Hanko S. M. Xiong C. Morris J. C. (2010). Unified Parkinson's disease rating scale-motor exam: inter-rater reliability of advanced practice nurse and neurologist assessments. J. Adv. Nurs. 66, 1382–1387. 10.1111/j.1365-2648.2010.05313.x 20546368 PMC2903978

[B53] Pavese N. Evans A. Tai Y. Hotton G. Brooks D. Lees A.. (2006). Clinical correlates of levodopa-induced dopamine release in Parkinson's disease: a pet study. Neurology 67, 1612–1617. 10.1212/01.wnl.0000242888.30755.5d 17101892

[B54] Pincus S. (1994). Singular stationary measures are not always fractal. J. Theor. Prob. 7, 199–208. 10.1007/BF02213368

[B55] Postuma R. B. Berg D. Stern M. Poewe W. Olanow C. W. Oertel W.. (2015). MDS clinical diagnostic criteria for Parkinson's disease. Mov. Disord. 30, 1591–1601. 10.1002/mds.26424 26474316

[B56] Rinne U. (1983). Problems associated with long-term levodopa treatment of Parkinson's disease. Acta Neurol. Scand. 68, 19–26. 10.1111/j.1600-0404.1983.tb01513.x 6587715

[B57] Rosqvist K. Hagell P. Odin P. Ekström H. Iwarsson S. Nilsson M. (2017). Factors associated with life satisfaction in Parkinson's disease. Acta Neurol. Scand. 136, 64–71. 10.1111/ane.12695 27726132

[B58] Schulz K. F. Altman D. G. Moher D. CONSORT Group. (2010). CONSORT 2010 statement: updated guidelines for reporting parallel group randomised trials. BMJ. 340, c332. 10.1136/bmj.c332 20332509 PMC2844940

[B59] Sehm B. Kipping J. A. Schäfer A. Villringer A. Ragert P. (2013). A comparison between uni-and bilateral tDCS effects on functional connectivity of the human motor cortex. Front. Human Neurosci. 7, 183. 10.3389/fnhum.2013.00183 23675337 PMC3646257

[B60] Shaheiwola N. Zhang B. Jia J. Zhang D. (2018). Using tDCS as an add-on treatment prior to FES therapy in improving upper limb function in severe chronic stroke patients: a randomized controlled study. Front. Human Neurosci. 12, 233. 10.3389/fnhum.2018.00233 29970994 PMC6018756

[B61] Tiffin J. Asher E. J. (1948). The purdue pegboard: norms and studies of reliability and validity. J. Appl. Psychol. 32, 234. 10.1037/h0061266 18867059

[B62] Timmer J. Gantert C. Deuschl G. Honerkamp J. (1993). Characteristics of hand tremor time series. Biol. Cybern. 70, 75–80. 10.1007/BF00202568 8312399

[B63] Tinazzi M. Del Vesco C. Fincati E. Ottaviani S. Smania N. Moretto G.. (2006). Pain and motor complications in Parkinson's disease. J. Neurol. Neurosurg. Psychiatry 77, 822–825. 10.1136/jnnp.2005.079053 16549416 PMC2117476

[B64] Tombaugh T. N. McIntyre N. J. (1992). The mini-mental state examination: a comprehensive review. J. Am. Geriatr. Soc. 40, 922–935. 10.1111/j.1532-5415.1992.tb01992.x 1512391

[B65] Tomlinson C. L. Stowe R. Patel S. Rick C. Gray R. Clarke C. E. (2010). Systematic review of levodopa dose equivalency reporting in Parkinson's disease. Mov. Disord. 25, 2649–2653. 10.1002/mds.23429 21069833

[B66] Valentino F. Cosentino G. Brighina F. Pozzi N. G. Sandrini G. Fierro B.. (2014). Transcranial direct current stimulation for treatment of freezing of gait: a cross-over study. Mov. Disord. 29, 1064–1069. 10.1002/mds.25897 24789677

[B67] van Nuland A. J. den Ouden H. E. Zach H. Dirkx M. F. van Asten J. J. Scheenen T. W.. (2020). Gabaergic changes in the thalamocortical circuit in Parkinson's disease. Human Brain Map. 41, 1017–1029. 10.1002/hbm.24857 31721369 PMC7267977

[B68] Wagner T. Fregni F. Fecteau S. Grodzinsky A. Zahn M. Pascual-Leone A. (2007). Transcranial direct current stimulation: a computer-based human model study. Neuroimage 35, 1113–1124. 10.1016/j.neuroimage.2007.01.027 17337213

[B69] Weintraub D. Stern M. B. (2005). Psychiatric complications in Parkinson disease. Am. J. Geriatr. Psychiatry 13, 844–851. 10.1097/00019442-200510000-00003 16223962

[B70] Zhang B. Huang F. Liu J. Zhang D. (2018). A novel posture for better differentiation between Parkinson's tremor and essential tremor. Front. Neurosci. 12, 317. 10.3389/fnins.2018.00317 29867328 PMC5966572

[B71] Zhang D. Poignet P. Widjaja F. Ang W. T. (2011). Neural oscillator based control for pathological tremor suppression via functional electrical stimulation. Control Eng. Pract. 19, 74–88. 10.1016/j.conengprac.2010.08.009

